# A Novel Volume-Age-KPS (VAK) Glioblastoma Classification Identifies a Prognostic Cognate microRNA-Gene Signature

**DOI:** 10.1371/journal.pone.0041522

**Published:** 2012-08-03

**Authors:** Pascal O. Zinn, Pratheesh Sathyan, Bhanu Mahajan, John Bruyere, Monika Hegi, Sadhan Majumder, Rivka R. Colen

**Affiliations:** 1 Department of Neurosurgery, Baylor College of Medicine, Houston, Texas, United States of America; 2 Department of Genetics, MD Anderson Cancer Center, University of Texas, Houston, Texas, United States of America; 3 Department of Radiology, MD Anderson Cancer Center, University of Texas, Houston, Texas, United States of America; 4 Department of Clinical Neurosciences, University Hospital (CHUV BH19-110), Lausanne, Switzerland; 5 Department of Radiology, Brigham and Women's Hospital, Harvard Medical School, Boston, Massachusetts, United States of America; The University of Chicago, United States of America

## Abstract

**Background:**

Several studies have established Glioblastoma Multiforme (GBM) prognostic and predictive models based on age and Karnofsky Performance Status (KPS), while very few studies evaluated the prognostic and predictive significance of preoperative MR-imaging. However, to date, there is no simple preoperative GBM classification that also correlates with a highly prognostic genomic signature. Thus, we present for the first time a biologically relevant, and clinically applicable tumor Volume, patient Age, and KPS (VAK) GBM classification that can easily and non-invasively be determined upon patient admission.

**Methods:**

We quantitatively analyzed the volumes of 78 GBM patient MRIs present in The Cancer Imaging Archive (TCIA) corresponding to patients in The Cancer Genome Atlas (TCGA) with VAK annotation. The variables were then combined using a simple 3-point scoring system to form the VAK classification. A validation set (N = 64) from both the TCGA and Rembrandt databases was used to confirm the classification. Transcription factor and genomic correlations were performed using the gene pattern suite and Ingenuity Pathway Analysis.

**Results:**

VAK-A and VAK-B classes showed significant median survival differences in discovery (P = 0.007) and validation sets (P = 0.008). VAK-A is significantly associated with *P53* activation, while VAK-B shows significant *P53* inhibition. Furthermore, a molecular gene signature comprised of a total of 25 genes and microRNAs was significantly associated with the classes and predicted survival in an independent validation set (P = 0.001). A favorable *MGMT* promoter methylation status resulted in a 10.5 months additional survival benefit for VAK-A compared to VAK-B patients.

**Conclusions:**

The non-invasively determined VAK classification with its implication of VAK-specific molecular regulatory networks, can serve as a very robust initial prognostic tool, clinical trial selection criteria, and important step toward the refinement of genomics-based personalized therapy for GBM patients.

## Introduction

Glioblastoma Multiforme (GBM) is the most common primary malignant brain tumor in adults. In the United States, more than 10,000 patients per year are newly diagnosed with GBM [1]. Despite existing multimodal treatment approaches, which normally include surgical resection followed by adjuvant radio-chemotherapy, the median overall survival remains at 14.6 months [2].

Despite our increasing knowledge of GBM molecular biology with the identification of GBM molecular subclasses and novel possibly targetable pathways [3], [4], [5], presurgical survival prediction is largely based on clinical factors such as age and KPS [6], [7], [8], [9], [10], [11], [12]. However, after invasive procedures and genomic data collection, extent of resection [7], [13], [14], [15] and molecular criteria, such as *O6-methylguanine-DNA-methyltransferase (MGMT)* promoter methylation, *isocitrate dehydrogenase 1 (IDH1)*, or glioma CpG island methylator phenotype (G-CIMP) status are significant prognostic and predictive criteria [7], [16], [17], [18].

With the recent upsurge of genomic discoveries and enhanced imaging technologies, in particular MRI, additional prognostic and predictive determinants for GBM patients are available. Previously, tumor volumes determined by MRI were shown to be a prognostic biomarker associated with survival in recurrent GBM [19]. Furthermore, MRI phenotypes of GBM tumors identified differential underlying molecular tumor compositions [20], [21], [22]. Patient stratification based on molecular characteristics has become increasingly important. As such, Hegi et al [2], [23] demonstrated a survival benefit in patients with methylation of the *MGMT* promoter treated with combined Temozolomide (TMZ) and radiotherapy, with median overall survival of 23.4 months compared with 12.6 months in the non-methylated group [2]. Etienne and colleagues demonstrated that older patients, who often have the De Novo (primary) form of GBM, have EGFR overexpression which is responsible for increased angiogenesis, edema, and invasion and might account for the decrease in survival in elderly patients [24]; younger patients more often exhibit a secondary form of glioblastoma that is associated with *TP53* mutation [24]. A recent study demonstrated that GBM can be divided into four molecular subgroups [25], although, no significant survival differences among the groups were observed.

Imaging has been shown to be able to non-invasively reflect underlying tumor biology and genomics [20], [21], [22], [26], thus, a simple classification which incorporates imaging could improve existing prognostic criteria in a clinically relevant way.

Therefore, in this study, we propose and validate a simple and highly prognostic GBM classification system which incorporates preoperative tumor volumetry along with age and KPS (VAK) that allows for non-invasive preoperative predictions at patient admission. We also determine the VAK associated cognate microRNA-gene regulatory networks inherent to each class which might allow for a class-specific therapeutic approach.

## Methods

The collection of the original material and data of The Cancer Genome Atlas (TCGA) and The Cancer Imaging Archive (TCIA) study was conducted in compliance with all applicable laws, regulations and policies for the protection of human subjects, and necessary IRB approvals were obtained [27]. The TCGA is a NCI sponsored publicly available resource which has produced a multi-dimensional genomic and clinical data set in GBM and other cancers [27]. Image data used in this research were obtained from TCIA (http://cancerimagingarchive.net/) sponsored by the Cancer Imaging Program, DCTD/NCI/NIH. This repository contains the imaging corresponding to the patients of the TCGA. GBM patients for the validation set were also obtained from the REpository for Molecular BRAin Neoplasia DaTa (REMBRANDT) [28].

### Patient population

We identified 78 GBM patients from TCGA for whom full annotation of Age, KPS, and MGMT methylation status, and corresponding pretreatment MR imaging was available in the TCIA. An independent validation dataset (N = 64) comparable to the discovery set with regard to lesion volume, age, KPS, gender, and survival distribution across VAK-A and VAK-B patients was collated using both the Rembrandt and TCGA/TCIA databases. The prognostic VAK derived cognate microRNA-gene signature was validated using an independent larger TCGA dataset (N = 255).

### Image Acquisition and Analysis

**Figure 1:**
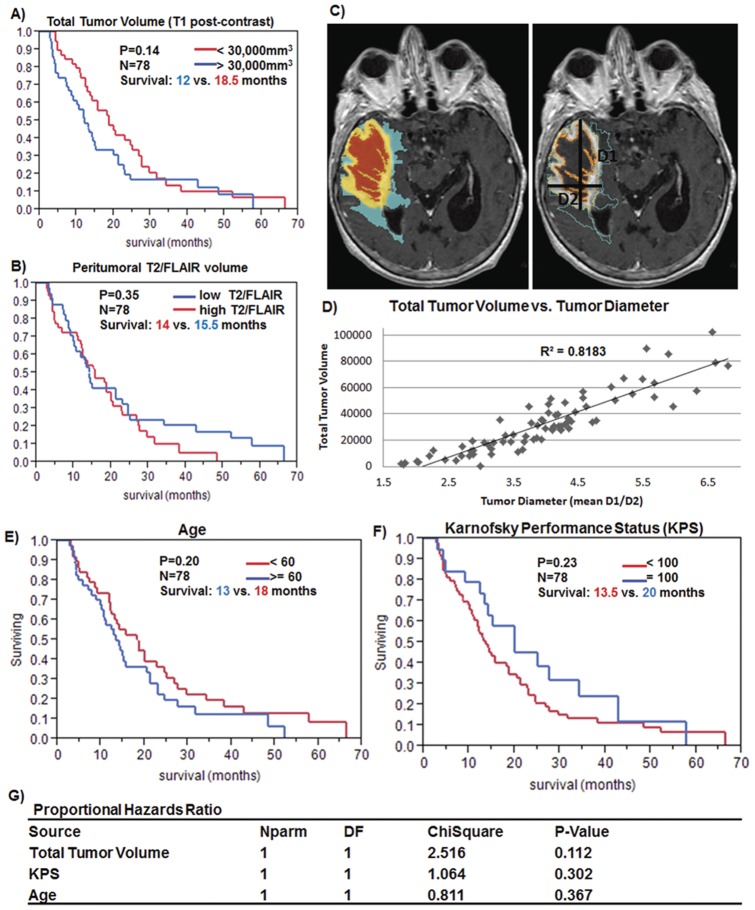
Volume, Age, KPS GBM patient survival. (**A**) Kaplan Meier analysis based on high versus low volumes of MRI T1 post-contrast images and (**B**) Kaplan Meier analysis based on high and low volumes of peritumoral MRI T2/FLAIR signal intensity. (**C**) Left panel: A segmented right temporal lobe glioblastoma is shown with volumes of necrosis (red), contrast enhancement (yellow) and edema/FLAIR (blue). (**C**) Right panel: Same patient as on left demonstrating how 2D measurements were performed. (**D**) Correlation of Total Tumor Volume and mean lesion diameter of the two largest orthogonal measures. Kaplan Meier survival analyses for (**E**) Age, (**F**) KPS and (**G**) Proportional Hazards Model for: Total Tumor Volume, Age, and KPS.

All images were downloaded from the TCIA. Image analysis was performed as previously described by our group [20]. In brief, 3D Slicer (www.slicer.org), an open-source software platform developed at our institution (Brigham and Women's Hospital, Harvard Medical School) for medical image processing and 3D visualization of image data, was used for tumor volumetry [29], [30], [31]; AIM Clear Canvas (http://www.clearcanvas.ca) was used to measure lesion diameters (mean of two largest orthogonal diameters (D1 and D2, Figure 1).

### Volume-Age-KPS (VAK) classification

The Volume, Age, and KPS (VAK) classification was created as follows. The tumor volume score was calculated for each patient. Patients were divided into groups based on the median lesion volume or lesion diameter (30,000 mm^3^/40 mm); those above the median cutoff were defined as high volume group and those below as low volume group. Patients were also stratified into 2 groups based on median age cutoff (those ≥60 years and <60 years) and KPS (those with KPS = 100 and KPS <100). A 3-point scoring system was used to dichotomize the patients into VAK-A and VAK-B groups (Table 1): Volume ≥30,000 mm^3^ or a lesion diameter ≥40 mm  = 1 point; Age ≥60 years  = 1 point, KPS <100 = 1 point. The sum of the latter three points defines the VAK classification. Those patients with 0 and 1 points were defined as VAK-A, while those with 2 and 3 points were defined as VAK-B (Table 1).

**Table 1:** VAK classification system.

3-point VAK classification system		
Volume ≥30,000mm^3^ or ≥40mm diameter	=	1 point
Age ≥60 years	=	1 point
KPS <100	=	1 point
VAK-A (good prognosis)	=	0 and 1 points
VAK-B (poor prognosis)	=	2 and 3 points

Methylation status (M) of the MGMT promoter was subsequently included in the VAK score to obtain the VAKM scores. VAKM patients were grouped into methylated (VAK + M) and non-methylated (VAK-M) groups.

**Table 2:** Gene and microRNA Ranking.

Gene and microRNA rank score based on product of 4 variables	
P-value	Differential expression across VAK-A/VAK-B classes
Fold Regulation	Differential fold regulation across VAK-A/VAK-B classes
Binding Strength	Number of algorithms predicting microRNA-gene bindings
Targeted Molecules	Number of inversely expressed molecules targeted within same class

### Genomic Analysis

Affymetrix level 1 mRNA, Agilent level 2 microRNA, and Level 3 Illumina and HumanMethylation27 methylation data were downloaded from the public TCGA data portal (October 2011) (http://cancergenome.nih.gov/). Affymetrix CEL file analysis was performed in R project, a free statistical computing software (http://www.r-project.org/) using the Bioconductor platform (http://www.bioconductor.org/). The Robust Multi-Array (RMA) algorithm was used for normalization [32]. To determine the MGMT promoter methylation status a median cutoff using the level 3 beta-value present in the TCGA was used (Illumina probes: An average of the following probes was used: MGMT_P272_R and MGMT_P281_F; Human Methylation probes (cg12434587 and cg12981137) were selected according to the recent publication by Bady et al [33].

In each patient, a total of 13,628 genes (22,267 hybridization probes) and 555 microRNAs (1,510 hybridization probes) were analyzed for significance and differential fold regulation in VAK-A and VAK-B classes by Comparative Marker Selection [34] (CMS) (Broad Institute, MIT, Cambridge, MA). The significant and inversely expressed genes and microRNAs in VAK-A and VAK-B were analyzed using Ingenuity Pathway Analysis (IPA) (www.ingenuity.com).

### microRNA Target Prediction and Rank Score

Potentially binding microRNAs for the top 100 VAK-A and VAK-B associated genes were determined using miRWalk [35], a database which predicts microRNA targets. For each molecule, a VAK gene- and microRNA rank score consisting of the product of four variables (P value, fold regulation, binding strength, and number of targeted molecules) was assigned (Table 2). This VAK rank score measures the degree of correlation of a molecule with either VAK class, how strong the microRNA potentially binds to an inversely correlated mRNA, and how many inversely correlated top ranking mRNAs or microRNAs are targeted by other high ranking molecules. A high ranking molecule is a highly targeted and targeting molecule that is VAK-A or VAK-B specific, thus, likely a strong regulator of the VAK-A or VAK-B phenotype. The rank score for microRNAs is negative and the rank score for mRNAs is positive since they are inversely expressed. The top genes and microRNAs for each class (total N = 37) were analyzed by IPA to generate the cognate VAK-A and VAK-B microRNA-gene regulatory networks.

### VAK-25-gene- and microRNA signature

Ingenuity Pathway Analysis revealed a highly connected network of a total of 25 molecules to form the cognate microRNA-gene networks associated with VAK-A (12 molecules) and VAK-B classes (13 molecules). To account for the inverse expression pattern and prognostic aspect of the genes and microRNAs, these 25 molecules were then combined into a single signature by using the ratio of the mean (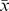) microRNA/gene expression of VAK-A/VAK-B molecules.


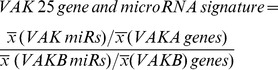


Median cutoff was used to separate the patients into significant survival groups based on the above VAK-25-gene- and microRNA signature in an independent TCGA validation set (N = 255).

### Ingenuity Transcription Factor Analysis

Ingenuity Pathway Transcription Factor Analysis was used to identify the transcription factors that may be responsible for gene expression changes observed in the VAK-A and VAK-B patient groups. IPA predicts which transcription factors are activated or inhibited to explain the upregulated and downregulated genes observed across the VAK groups. The transcriptional regulation is measured by the z-score. The basis for z-score predictions are relationships in the molecular pathways (networks). The relationships represent experimentally observed gene expression or transcription events and they must be associated with a direction of change that is either activating or inhibiting (as derived from the literature compiled in the Ingenuity® Knowledge Base). A z-score of below or above 2 is considered significant.

### Statistical Analysis

Kaplan-Meier method and log-rank test were used to compute overall median survival. Cox proportional hazards likelihood ratio was used for direct comparison and relative survival effects of the examined VAK variables. The statistical analyses were performed using Microsoft Excel 2010 and JMP Pro 10.0 (SAS, Cary NC) software packages.

## Results

**Figure 2:**
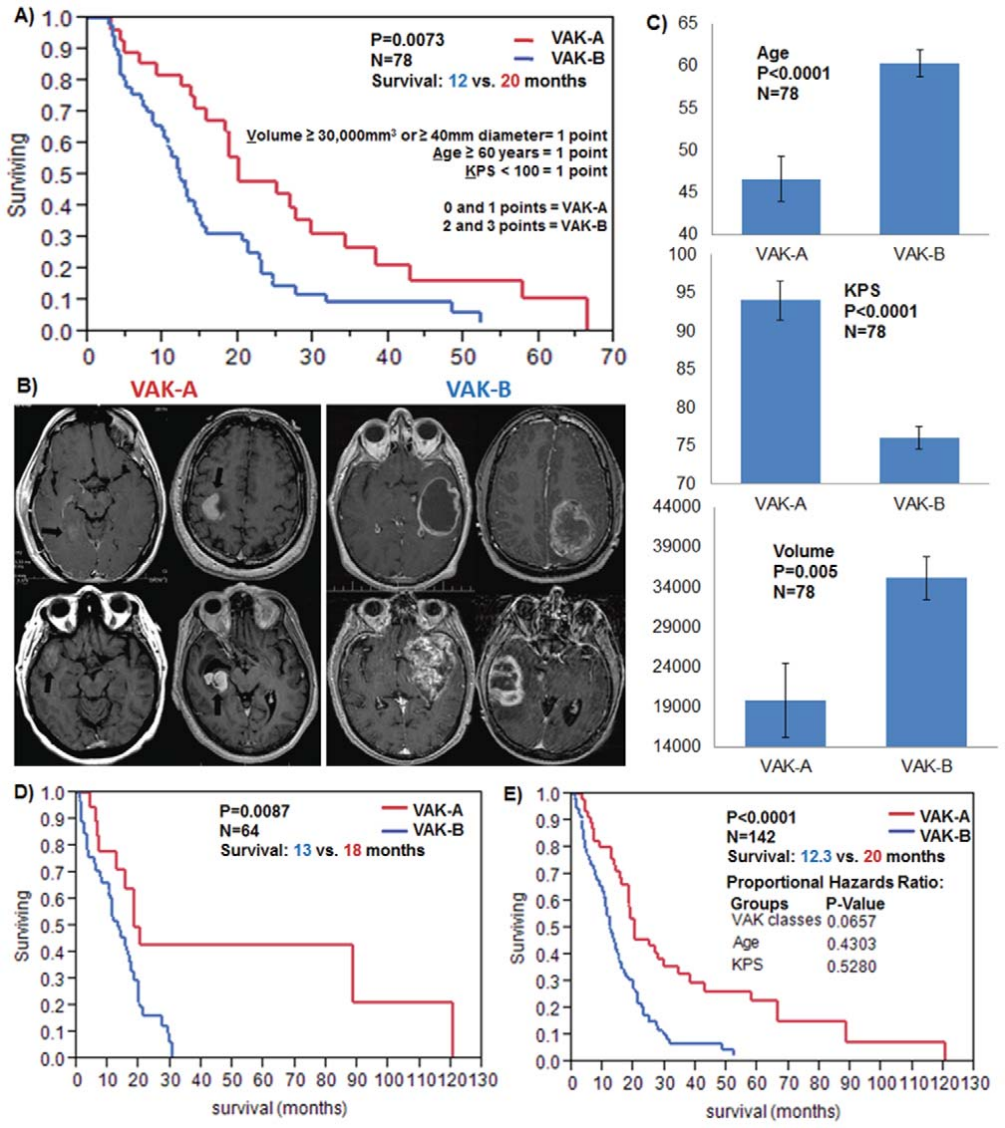
Volume, Age, KPS (**VAK**) **classification and phenotype.** **V**olume, **A**ge, **K**PS (VAK)-A and B classes showing (**A**) Kaplan Meier survival plot (**B**) representative MRI images for VAK-A and VAK-B patients and (**C**) Mean volume, Age, and KPS in VAK-A and VAK-B classes. (**D**) VAK-A and VAK-B survival validation in an independent patient set (N = 64) and (**E**) combination of the discovery and validation set (N = 142) for patient with full VAK annotaiton including the Proportional Hazards Model correcting for Age and KPS.

In order to create a non-invasive simple GBM classification that can be determined upon patient admission in every clinical setting, we compared the prognostic significance of the two most frequently used MRI sequences, T1 post-contrast and T2/FLAIR. Our data show that the total tumor volume corresponding to T1 post-contrast enhancing and necrotic tumor parts is a stronger prognostic criterion than the volume of peritumoral T2/FLAIR signal when using a median cutoff (Figures 1A and 1B, Table S1). Stratification of GBM patients based on their total tumor volumes (contrast enhancement + necrosis) or tumor diameter yielded a trend toward longer survival with smaller lesions (P = 0.14, Survival: 12 vs. 18.5 months) when considering median cutoff (30,000 mm^3^) as previously described in the literature [36]. The mean of the largest two orthogonal diameters of the lesion highly correlated with the total tumor volume (R^2^ = 0.82, Figures 1C and 1D, Table S1) and thus a standard 2D lesion diameter could be used as a surrogate for tumor volume. We further hypothesized that by utilizing additional known prognostic factors we could augment the prognostic value of either variable by creating a simple classification. Similar as for tumor volume, we analyzed two additional variables: Age and Karnofsky Performance Status (KPS). Both Age and KPS independently did not yield significance (Age: P = 0.2, Survival: 13 vs. 18 months; KPS: P = 0.23, Survival: 13.5 vs. 20 months) (Figures 1E, 1F, and 1G, Table S1). However, when we combined Volume, Age, and KPS to form the VAK classification using an easy-to-use 3-point system, we achieved a significant survival segregation of the patient population (P = 0.007, Survival: 12 vs. 20 months) (Figure 2A, Table 1, Table S2). We termed the two patient groups VAK-A and VAK-B, whereas VAK-A demonstrated a favorable and VAK-B an unfavorable prognostic significance (Figure 2, Table S2). As expected, tumor volume, age, and KPS were significantly associated with either VAK-A and VAK-B classes (Figures 2B and 2C). For validation, we collated a VAK confirmation set consisting of patients (N = 64) from both the Rembrandt and TCGA databases that were not used in the discovery set (P = 0.0087, Survival: 13 vs. 18 months) (Figure 2D, Table S2). A merge of discovery and validation sets yielded a VAK-A survival advantage of 20 versus 12.3 months in 142 patients (P<0.0001) while the VAK classification clearly was superior when corrected for Age and KPS (Figure 2E, Table S2).

**Figure 3:**
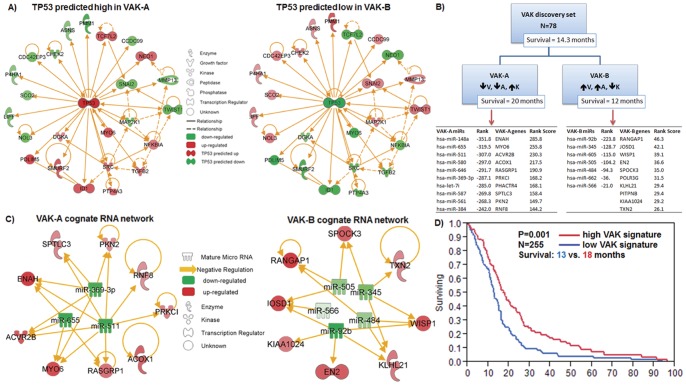
VAK molecular characterization. (**A**) *TP53* activation and inhibition across VAK-A and VAK-B patient classes together with molecular regulatory networks of differentially regulated genes. (**B**) VAK-A and VAK-B classes with corresponding top differentially expressed genes- and microRNAs and (**C**) graphical representation of the prognostic cognate gene- and microRNA VAK-A and VAK-B networks consisting of a total of 25 molecules. (**D**) Kaplan Meier survival plot using the VAK-derived 25 gene- and microRNA signature in an independent TCGA validation set of 255 patients.

We then sought to determine the VAK associated molecular tumor configuration. To this end, the most significantly differentially regulated genes across VAK-A and VAK-B were identified and transcription factor analysis predicted the tumor suppressor *P53* to be the only transcriptional regulator significantly activated in VAK-A, while inactivated in VAK-B (z-score  =  +/−2.7 respectively) (Figure 3A, Table S3). For additional molecular characterization of VAK, we identified the VAK-A and VAK-B cognate gene and microRNA networks potentially contributing to the observed survival difference. We assigned a combined rank score to every molecule. The rank score consisted of the following variables: Class-specific inverse microRNA-gene fold regulation, P value, number of targeted and significant inversely expressed molecules, and average binding predictions of the targeted or targeting molecules (Figure 3B, Table 2). The high positive or high negative rank scores thus reflect the positive or negative correlation and the VAK class specific importance of a molecule (Figure 3B and 3C). The top inversely correlated VAK-A and VAK-B predicted genes and microRNAs (Figure 3B) analyzed by IPA resulted in 25 molecules, while 12 molecules (3 microRNAs, 9 mRNAs) fell into VAK-A and 13 molecules (5 microRNAs, 8 mRNAs) fell into VAK-B (Figure 3C).

**Figure 4:**
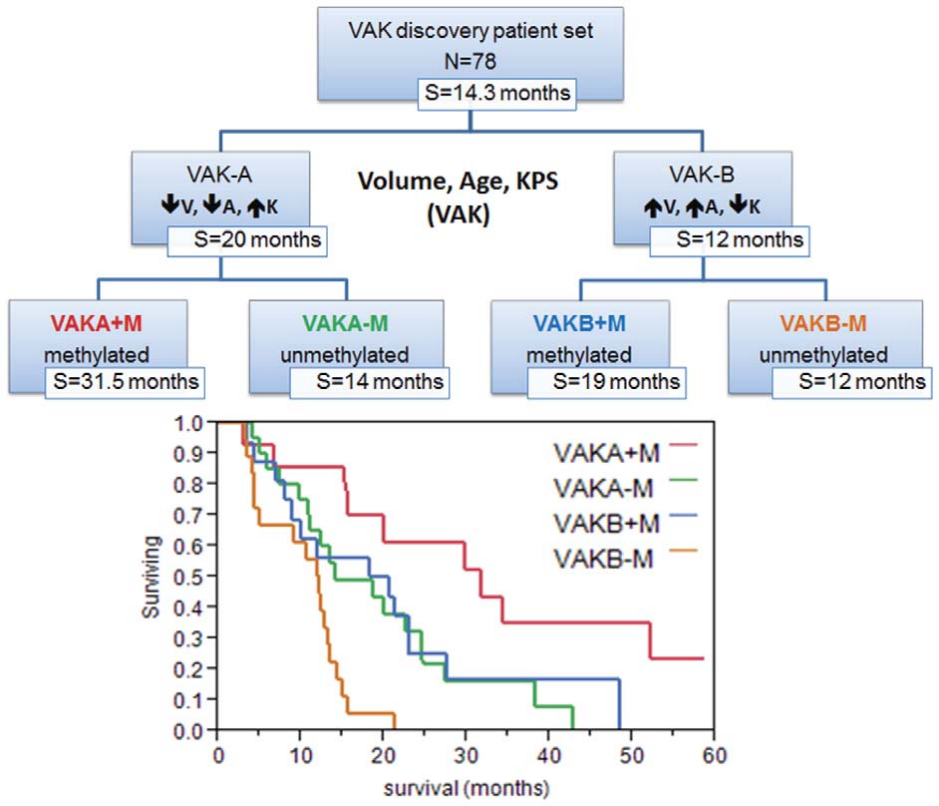
VAK MGMT model. Refined VAK classification with MGMT promoter methylation stratification, demonstrating the increased survival benefit for VAK-A with favorable MGMT status.

In order to validate the prognostic power of the 25 VAK-derived molecules, we collated an independent larger TCGA gene- and microRNA expression set (N = 255 patients) and applied the VAK-derived 25 gene- and microRNA signature (Figure 3D, Table S4) by means of the gene signature equation detailed in the methods. When we considered a median cutoff using the 25-gene- and microRNA signature, the 255 patients were segregated into significantly different survival groups (P = 0.001, Survival: 13 vs. 18 months) (Figure 3D, Table S4). When considering MGMT status with the VAK-A and VAK-B patient groups, our data show that a favorable MGMT promoter methylation status (discovery set, N = 78) resulted in a 10.5 months median survival advantage for VAK-A compared to VAK-B patients (Figure 4). Interestingly, VAK-B patients' MGMT status yielded only a 1 month survival difference both with and without TMZ treatment, while VAK-A patients appear to benefit from a favorable MGMT status both with (7.5 months) and without (3 months) Temozolomide therapy (Figure S1).

The 25-gene- and microRNA signature together with available annotation of Age and KPS in the 255 patient dataset also yielded two very distinct survival groups (P<0.0001, Survival: 11.5 vs. 18.5 months) (Figure S2A, Table S5). Analysis of the gene signature's prognostic significance together with age, and KPS with changing MGMT promoter methylation status yielded similar results as for the volume, age, and KPS classification (Figure S2B, Table S6). When analyzing survival differences of the continuous VAK score, such as VAK 0, 1, 2, and 3 points, we observe a point dependent decrease in median survival (24.9, 20, 13, and 10.5 months respectively), whereas 0 and 1 points, as well as 2 and 3 points cluster into two distinct survival groups, thus, supporting the dichotomized VAK-A (0 and 1 points) and VAK-B (2 and 3 points) classification (Figure S3).

Thus, VAK establishes an initial non-invasive GBM patient stratification with clinical and molecular significance.

## Discussion

Survival in newly admitted presurgical patients has most often been predicted by clinical factors, most commonly Age and KPS^,^ [6], [7], [9], [10], [12]. Nevertheless, here we show that preoperative MRI based total tumor volume is of stronger prognostic significance as shown by the proportional hazards test (Figure 1G) and, when paired with age and KPS, creates a robust survival classification for all preoperative GBM patients with availability of tumor volume, age, and KPS annotation (Figures 1 and 2). A frequently used prognostic GBM and Anaplastic Astrocytoma classification is the Recursive Partitioning Analysis (RPA) [37]. A direct comparison to VAK is limited as RPA requires additional variables such as surgical resection, neurological and mental status to allow for prognostic classification of all patients. These variables are not present in the TCGA dataset. However, when we create a 3-tier VAK classification to match the three RPA classes in the same dataset where RPA annotation is available, we see a more significant median survival difference between the extremes of VAK groups relative to the RPA classification (Figure S4). This underscores the fact that preoperative volume should be considered in initial prognostic predictions together with age and KPS in GBM patients. When comparing MGMT promoter methylation status across VAK-A and VAK-B; our data demonstrates that VAK-A patients take more advantage of both a favorable MGMT methylation status and Temozolomide therapy (Figure 4, Figures S1 and S2). This significant survival difference observed in VAK-A patients may occur due to the longer survival in general and thus prolonged exposure to therapy, however, even in the absence of TMZ, VAK-A patients with a favorable MGMT status show a 3.5 fold increased median survival compared to VAK-B patients, although 35% of the Temozolomide untreated group still received radiotherapy, compared to 95% of the Temozolomide treated group (Figure S1, left panel). This potentially demonstrates a unique underlying molecular tumor composition in VAK-A and VAK-B GBM patients which is further corroborated by the distinct genomic signature of both gene- and microRNA cognate networks inherent to VAK-A and VAK-B. More importantly, the VAK-derived 25-gene- and microRNA signature is significantly prognostic in an independent validation set and represents an independently significant variable together with age and KPS in the proportional hazards test. The fact that the latter three variables are independent and significant prognostic criteria is an additional rationale for the strength of a combined VAK-genomic classification. Whole genome expression- and transcription factor analysis predicts *P53* to be significantly activated in VAK-A, while inactivated in VAK-B patients (Figure 3A; Table S3). The ability to preoperatively predict *P53* activation status based on the proposed non-invasive and simple classification supports the association of VAK-A and VAK-B with distinct underlying molecular signatures. Furthermore, VAK-A predominantly contains down-regulated oncomirs [38], [39], [40] binding to up-regulated tumor suppressor genes [41], [42], [43], while VAK-B predominantly contains down-regulated tumor suppressor microRNAs [44], [45], [46] binding to up-regulated oncogenes [46], [47], [48], [49], [50] (Figure 3C). Taken together, this suggests that the VAK prognostic classification carries a unique molecular configuration driving or inhibiting oncogenesis in GBM.

This classification is important as it demonstrates that an initial patient classification based on very robust survival variables such as tumor volume, age, and KPS (VAK) can be determined upon patient admission in any clinical setting capable of MRI and should be considered a first step. Interestingly, we found that the tumor volume was highly correlated (R^2^ = 0.82, Figure 1D) with the tumor diameter and thus can be used as an accurate marker for the tumor volume. This is important as volumetric measurements require more time and manpower, while tumor diameter is easy to obtain and can be done by the radiologist or treating clinician at the imaging workstation. As more refined clinical and molecular variables become available during the treatment course, class refinement in addition to the VAK classification can yield more accurate insights into the role of potential targetable and survival/therapy predictive molecular factors. In particular, based on our data one can hypothesize that a combined classification using VAK together with the VAK-derived 25-gene- and microRNA signature and MGMT status can have a very robust prognostic and predictive significance for GBM patients. Furthermore, the VAK classification has the potential to facilitate patient selection for clinical trials. As an example patients with VAK score 2 and 3 (VAK-B class) having a poor survival and small benefit from multimodal therapy and favorable MGMT status (Figure 4, Figures S1 and S2), can be selected as candidates for new clinical trials rather than selecting the standard recurrent tumor patient group. The gene- and microRNA class stratification offered by the VAK classification can allow for novel Temozolomide and radiotherapy regimens based on the relative survival gain across VAK-A and VAK-B patient groups, or shed insight to possibly future agents which can be specifically developed to target the molecular signature of either VAK-A or VAK-B patients. Taken together, this suggests that the VAK classification is prognostic and predictive in nature.

Despite the strong evidence across discovery and validation sets, prospective validation of the joint VAK clinical/molecular classification is needed to confirm the potential therapeutic significance. However, the VAK classification with its implication of VAK-specific molecular regulatory networks, can serve as a very robust prognostic tool, clinical trial selection criteria, and important step toward the refinement of personalized therapy for GBM patients.

## Supporting Information

Figure S1 **VAK classification with and without MGMT methylation** (**VAKM**) **in the presence and absence of Temozolomide therapy.**
(TIF)

Figure S2 **VAK-derived 25 gene- and microRNA signature clinical significance** (**A**) **Kaplan Meier survival plot using the VAK-derived 25 gene- and microRNA signature together with Age and KPS in an independent TCGA set of 255 patients.** (**B**) Refined VAK classification by introducing MGMT promoter methylation by using the VAK-derived gene- and microRNA signature together with Age and KPS in a larger independent dataset (N = 255).
(TIF)

Figure S3 **Kaplan Meier survival based on the continuous VAK score, demonstrating the median survival decrease with a higher VAK score.**
(TIF)

Figure S4 **Comparison of the 3-tier-VAK and RPA survival classification.**
(TIF)

Table S1 **Calculations for Kaplan Meier plots for Total Tumor Volume, FLAIR signal, KPS, and age.**
(TIF)

Table S2 Calculations for VAK-A and VAK-B Kaplan Meier survival for the discovery, validation, and combined discovery and validation sets.
(TIF)

Table S3 **Ingenuity Pathway Analysis z-scores for** ***TP53*** **and other transcriptional regulators in VAK-A and VAK-B classes.**
(TIF)

Table S4 **Calculations for Kaplan Meier survival corresponding to the VAK-25-gene- and microRNA signature in an independent TCGA data set** (**N = 255**)**.**
(TIF)

Table S5 **Calculations for Kaplan Meier survival with age, KPS, and VAK-25-gene- and microRNA signature in the independent TCGA data set** (**N = 255**) **and proportional hazards ratio showing independent prognostic significance for the latter three variables.**
(TIF)

Table S6 **Calculations for VAKM Kaplan Meier survival for the discovery and the independent larger TCGA data set.**
(TIF)
