# Supplementary figures and images for: A Novel Volume-Age-KPS (VAK) Glioblastoma Classification Identifies a Prognostic Cognate microRNA-Gene Signature
Source: PLoS One. 2012 Aug 3;7(8):e41522. doi: 10.1371/journal.pone.0041522 (PMC3411674; doi:10.1371/journal.pone.0041522)

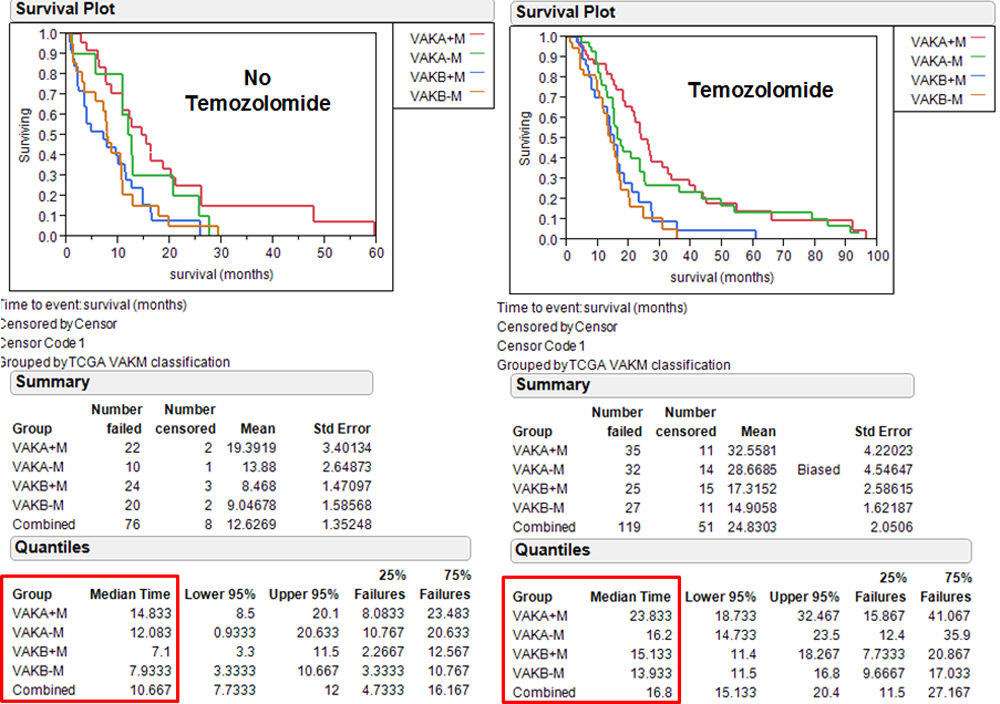

Supplement: Figure S1 — VAK classification with and without MGMT methylation (VAKM) in the presence and absence of Temozolomide therapy. (TIF) [file pone.0041522.s001.tif]

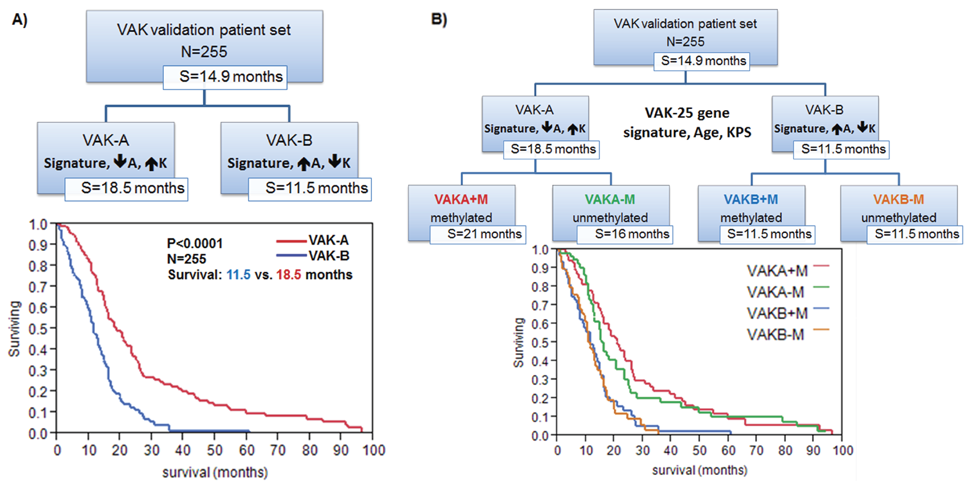

Supplement: Figure S2 — VAK-derived 25 gene- and microRNA signature clinical significance (A) Kaplan Meier survival plot using the VAK-derived 25 gene- and microRNA signature together with Age and KPS in an independent TCGA set of 255 patients. (B) Refined VAK classification by introducing MGMT promoter methylation by using the VAK-derived gene- and microRNA signature together with Age and KPS in a larger independent dataset (N = 255). (TIF) [file pone.0041522.s002.tif]

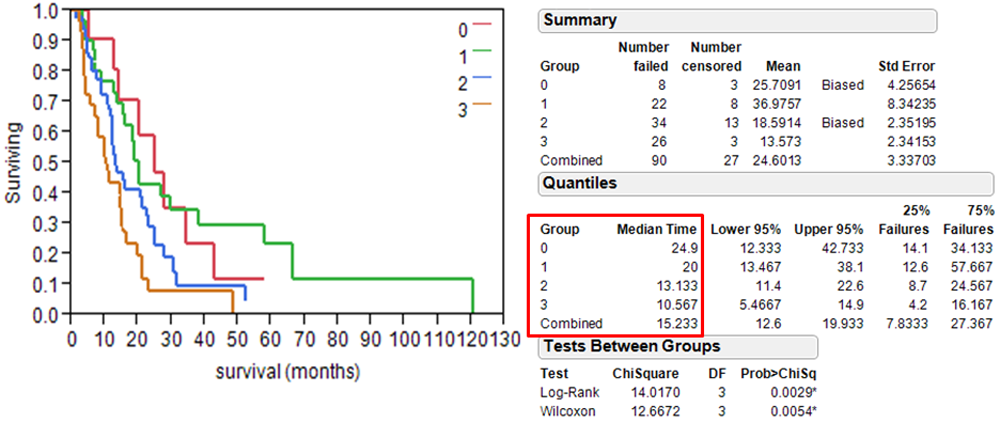

Supplement: Figure S3 — Kaplan Meier survival based on the continuous VAK score, demonstrating the median survival decrease with a higher VAK score. (TIF) [file pone.0041522.s003.tif]

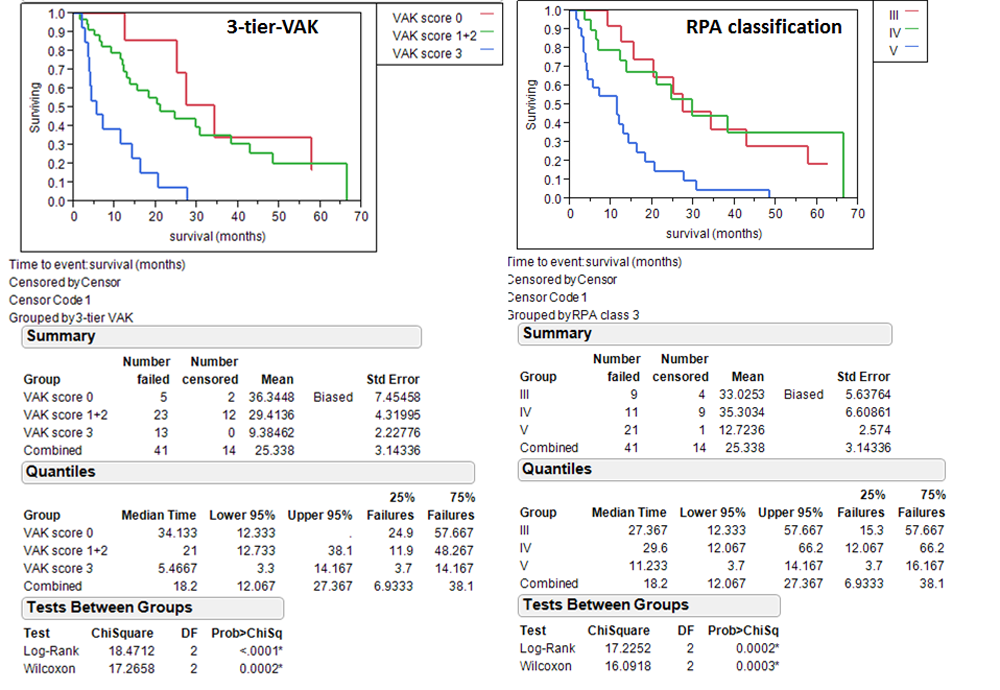

Supplement: Figure S4 — Comparison of the 3-tier-VAK and RPA survival classification. (TIF) [file pone.0041522.s004.tif]

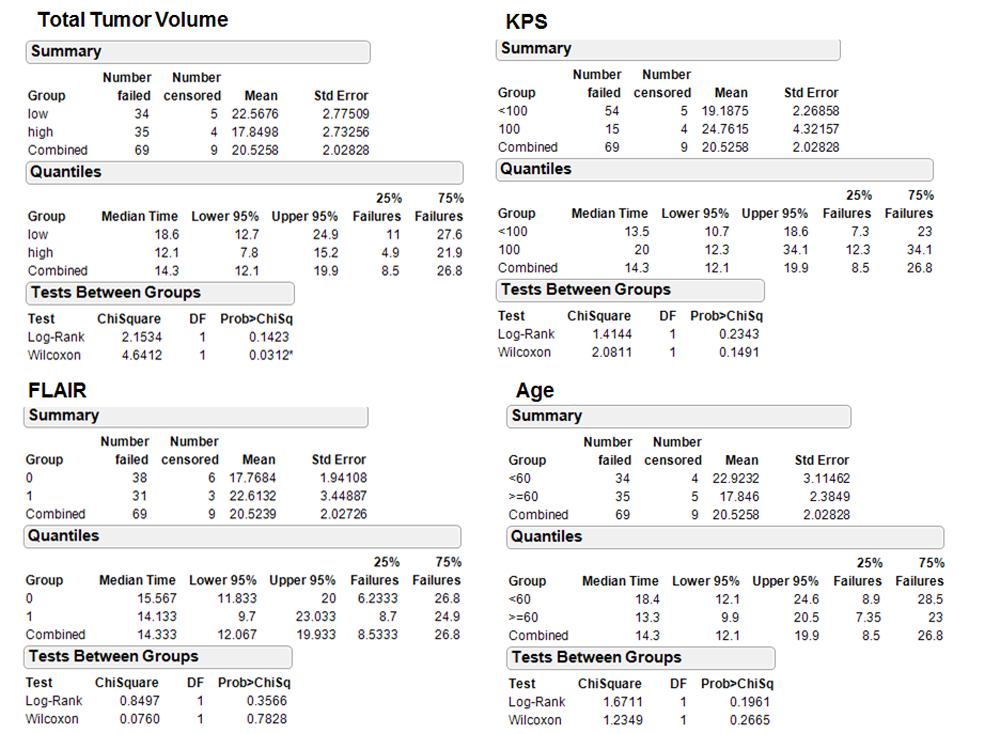

Supplement: Table S1 — Calculations for Kaplan Meier plots for Total Tumor Volume, FLAIR signal, KPS, and age. (TIF) [file pone.0041522.s005.tif]

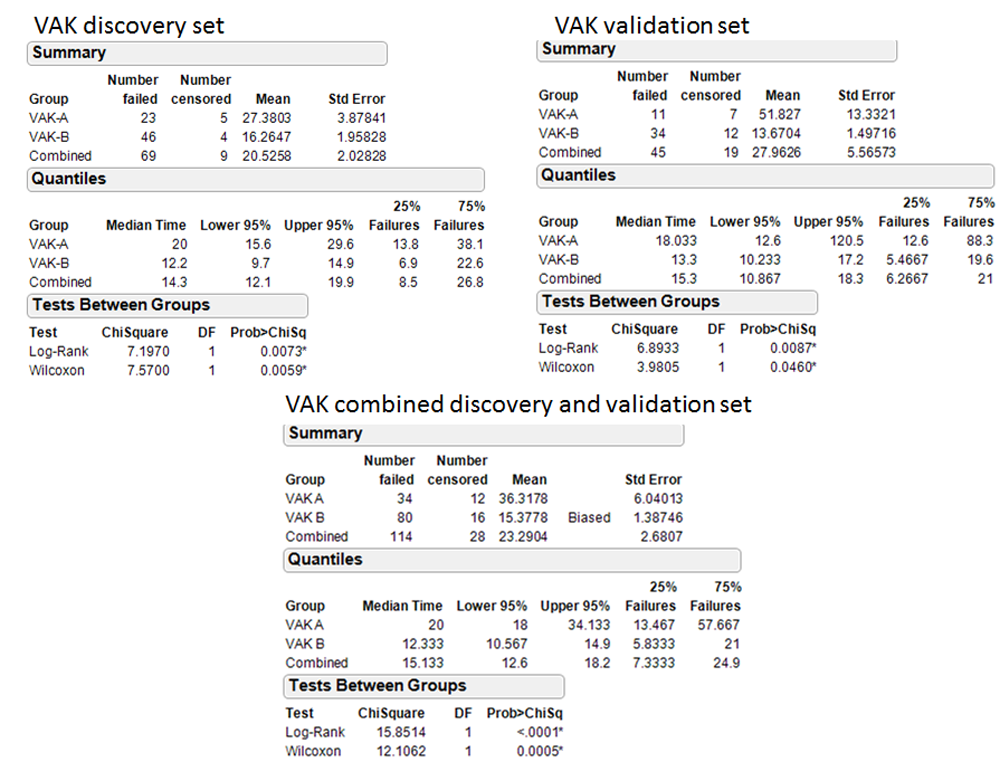

Supplement: Table S2 — Calculations for VAK-A and VAK-B Kaplan Meier survival for the discovery, validation, and combined discovery and validation sets. (TIF) [file pone.0041522.s006.tif]

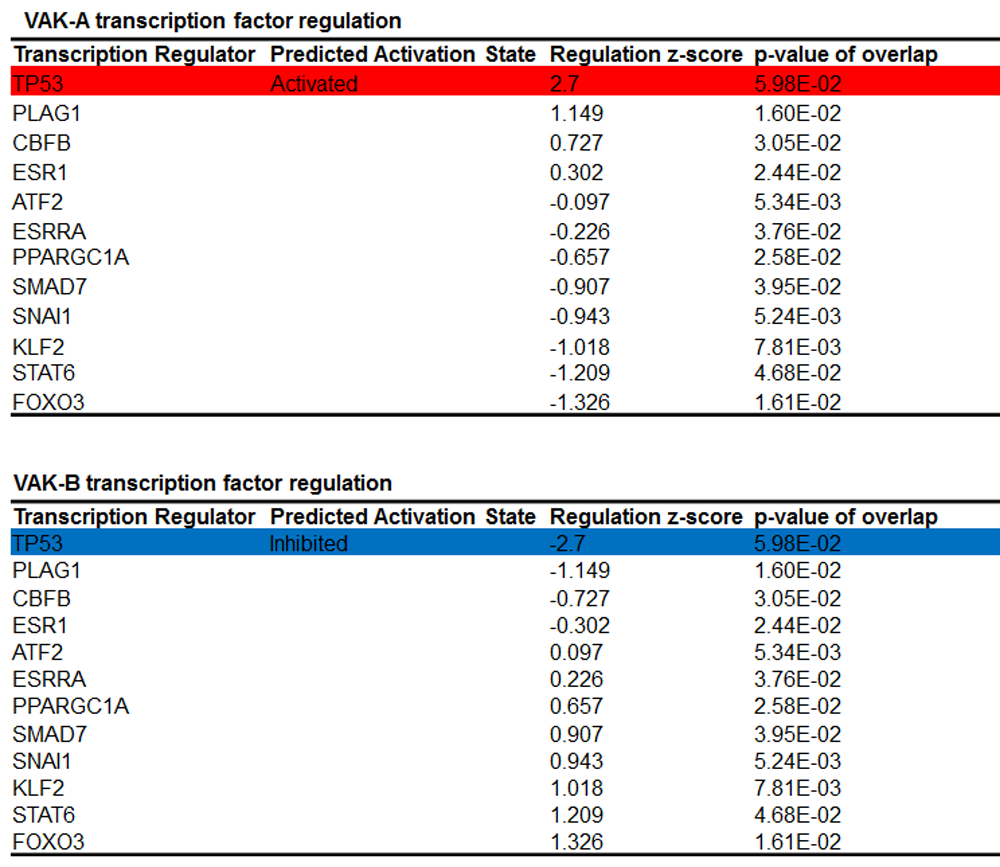

Supplement: Table S3 — Ingenuity Pathway Analysis z-scores for TP53 and other transcriptional regulators in VAK-A and VAK-B classes. (TIF) [file pone.0041522.s007.tif]

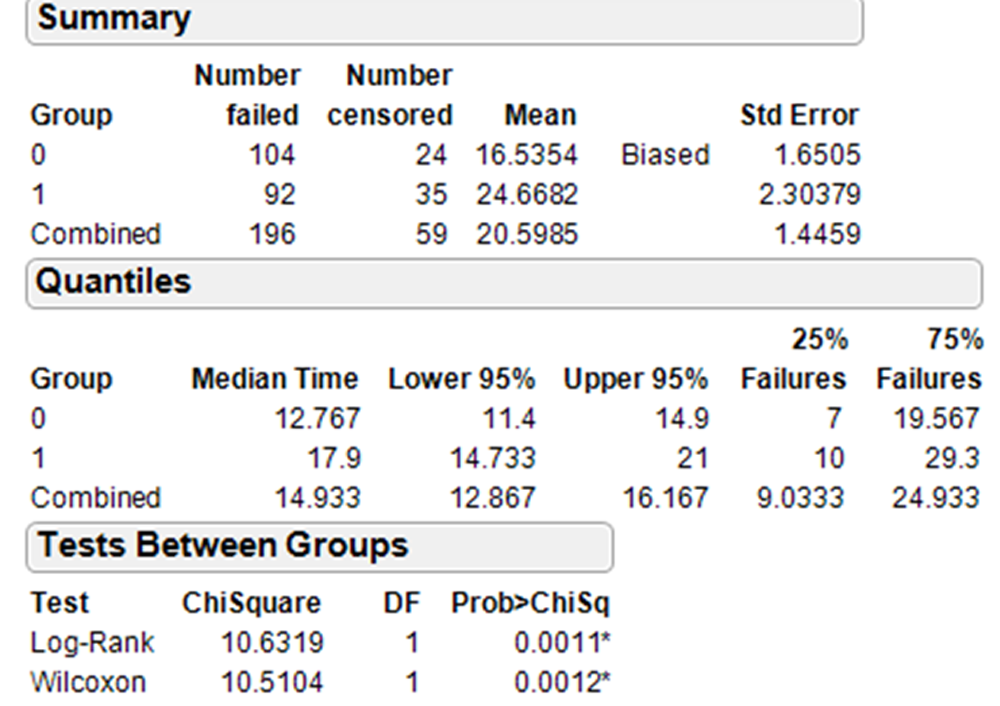

Supplement: Table S4 — Calculations for Kaplan Meier survival corresponding to the VAK-25-gene- and microRNA signature in an independent TCGA data set (N = 255). (TIF) [file pone.0041522.s008.tif]

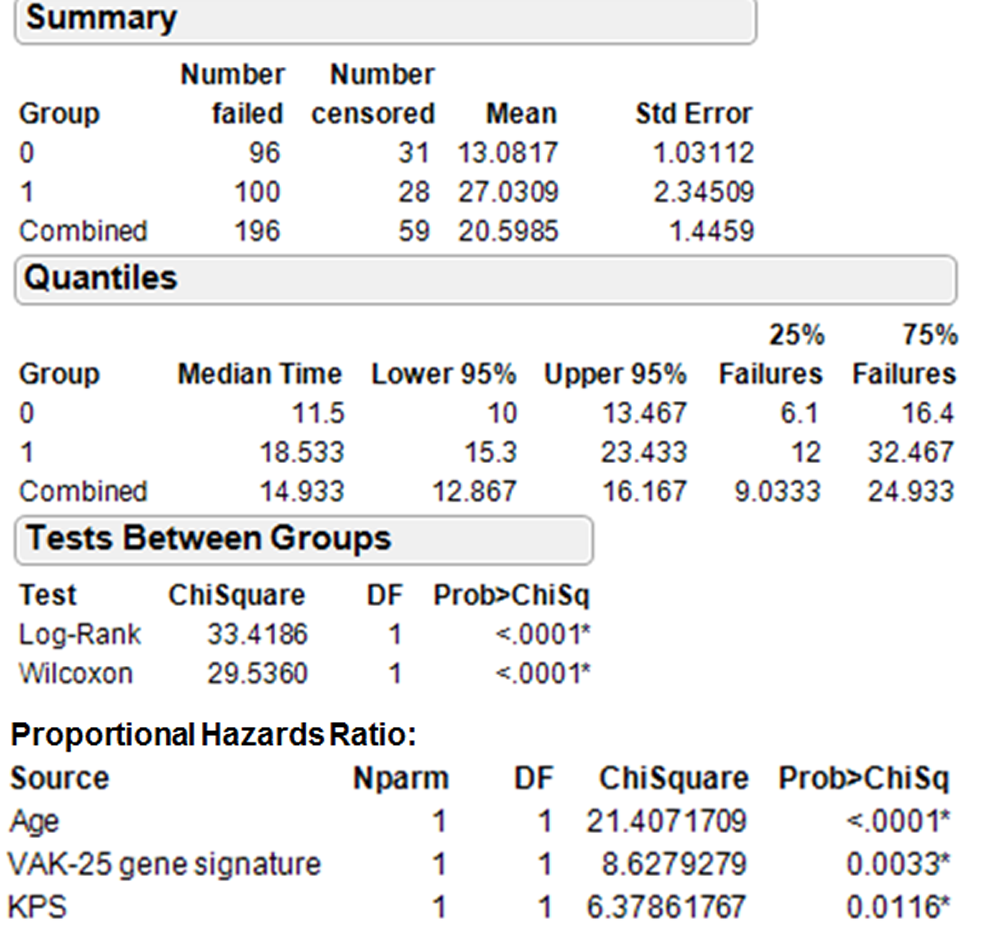

Supplement: Table S5 — Calculations for Kaplan Meier survival with age, KPS, and VAK-25-gene- and microRNA signature in the independent TCGA data set (N = 255) and proportional hazards ratio showing independent prognostic significance for the latter three variables. (TIF) [file pone.0041522.s009.tif]

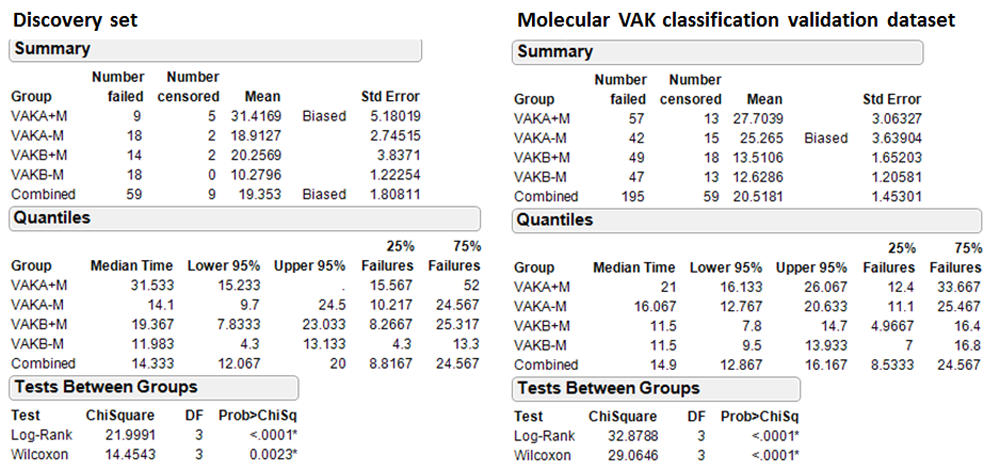

Supplement: Table S6 — Calculations for VAKM Kaplan Meier survival for the discovery and the independent larger TCGA data set. (TIF) [file pone.0041522.s010.tif]
